# Intercropping Flowering Plants Enhances Multiple Ecosystem Services in Apple Orchards

**DOI:** 10.3390/plants15142181

**Published:** 2026-07-16

**Authors:** Xingrui Zhang, Guodong Han, Yunli Xiao, Feng Ge

**Affiliations:** 1 Shandong Key Laboratory for Green Prevention and Control of Agricultural Pests, Institute of Plant Protection, Shandong Academy of Agricultural Sciences, Jinan 250100, China; xy20126043@163.com (X.Z.);; 2 Key Laboratory of Oasis Agricultural Pest Management and Plant Protection Resource Utilization of Xinjiang Uygur Autonomous Region, College of Agriculture, Shihezi University, Shihezi 832003, China; 3 Shandong Agricultural Technology Extension Center, Jinan 250100, China

**Keywords:** arthropods, pollination, pest control, soil fertility, fruit production

## Abstract

Intercropping flowering plants is an ecofriendly and sustainable orchard management practice, yet its effects on multiple ecosystem services remain poorly understood. To address this knowledge gap, we conducted a two-year field experiment using four species of intercropped flowering plants, including Sechuangzi *Cnidium monnieri* (L.) Cusson (Apiaceae), Japanese catnip *Schizonepeta tenuifolia* Briq. (Lamiaceae), rapeseed *Brassica napus* L. (Brassicaceae), and hairy vetch *Vicia villosa* Roth. (Fabaceae), in an apple orchard. We selected 22 ecosystem service indicators to establish a comprehensive framework for assessing orchard ecosystem services. In 2020, 19 indicators differed significantly between intercropped flowering plant plots and clean-tillage control plots, while 18 indicators showed significant differences in 2021. The ecosystem service indicators were grouped into six composite multifunctional indicators representing pollination, pest control, habitat provisioning, food provisioning, nutrient cycling, and carbon stock, all of which showed significant enhancement in flowering plant plots compared with the controls. Crucially, these six composite indices demonstrated synergistic interactions, with no trade-offs observed. Compared to clean-tillage control plots, total ecosystem services in 2020 increased by 228% in *C. monnieri* plots, 222% in *B. napus* plots, 217% in *V. villosa* plots, and 126% in *S. tenuifolia* plots; in 2021, the increases were 248%, 214%, 209%, and 115%, respectively. Overall, these results suggest that intercropping selected flowering plants can improve multiple measured ecosystem service indicators in apple orchards. This practice may provide a promising orchard-floor management strategy for enhancing ecosystem service multifunctionality under the conditions of this two-year field experiment.

## 1. Introduction

Modern intensified and homogenized agriculture relies on widespread use of chemical pesticides and large-scale monoculture, which have caused habitat loss, land degradation, greenhouse gas emissions, and harm to natural enemies and pollination processes [1,2]. To achieve sustainable agricultural development, practical changes are needed that safeguard ecosystem diversity, mitigate adverse environmental effects, and increase ecological services beyond agricultural output [3]. With such a shift, there is a transition from production-centered agricultural practices, focused solely on crop production, to more diversified management practices that support a broader range of ecosystem services [4].

Sustainable orchard management has great potential to enhance a variety of ecosystem services, leading to increased attention from various stakeholders [5]. Such management approaches primarily encompass several key practices aimed at enhancing ecosystem services, including increasing crop diversity through methods such as intercropping and crop rotation; promoting the diversity of non-crop species within or around orchards by establishing flower strips, shrub hedges, and seminatural habitats; introducing beneficial soil microorganisms, such as arbuscular mycorrhizal fungi, nitrogen-fixing bacteria, and growth-promoting bacteria; and minimizing soil disturbance, particularly through no-tillage methods [6,7]. These approaches combine biodiversity in agricultural landscapes with ecological principles in crop management to support the ecological intensification of agriculture [8].

Orchards are ideal agricultural landscapes for the implementation of sustainable ecological management [9]. Compared with arable and vegetable crops, orchards are perennial, implying increases in stability and resilience [10,11]. In addition, gaps are intentionally maintained between rows of fruit trees, and orchard perimeters are cultivated with plants with unique functional attributes. Orchards not only provide high-quality fruits but also have substantial capacity for carbon storage, similar to that of forests [12]. Intensive production in orchards relies on heavy pesticide usage and the extensive use of inorganic fertilizers, which have led to a series of problems such as increases in pest resurgence, pest resistance to pesticides, and pesticide residues, reductions in wild pollinators, and degradation of soil fertility [13,14]. Furthermore, orchards that incorporate non-crop elements, such as flower strips, hedges, and cover crops, benefit from increased plant diversity and enhanced ecosystem services, which in turn help reduce the reliance on synthetic pesticides and fertilizers [15]. Thus, intercropping annual or perennial flowering plants between the rows of fruit trees is an ecofriendly and sustainable practice of orchard management that deserves further study [16,17].

Flowering plants contribute to various ecosystem services within orchard systems through their interactions with fruit trees, affecting regulatory and provisioning services via aboveground and belowground pathways [15,18]. Different intercropped species can contribute to multiple ecosystem services. Sechuangzi *Cnidium monnieri* (L.) Cusson (Apiaceae) and Japanese catnip *Schizonepeta tenuifolia* Briq. (Lamiaceae) can enhance biological control by providing floral resources and habitat for natural enemies, while these species and other members of the Apiaceae and Lamiaceae families can also attract pollinators [14,19,20,21]. Rapeseed *Brassica napus* L. (Brassicaceae) provides abundant floral resources for pollinators, while *B. napus* and other members of the Brassicaceae family can also attract aphidophagous hoverflies and function as trap crops or insectary plants in pest management [22,23,24,25]. Hairy vetch *Vicia villosa* Roth. (Fabaceae) attracts pollinators [26] and supports natural enemies [19,21], while improving soil fertility by enhancing soil nitrogen availability and organic matter inputs [27,28,29,30]. Aboveground, flowering plants provide resources such as shelter, nectar, alternative prey/hosts, and pollen, which support beneficial arthropods. These resources increase fecundity, longevity, and survival, leading to increases in the population size and functions of natural enemies and pollinators [21,31]. Belowground, inputs of root exudates and flowering plant residues affect soil moisture, nutrients, structure, enzymes, and microbial communities [32]. However, intercropping flowering plants in orchards has rarely been studied for its effects on biodiversity and a range of ecosystem services. Consequently, there is a lack of a comprehensive understanding of how this practice affects biodiversity and various ecosystem services, including crop yield [33]. Ecosystem services contribute significantly to human welfare, both directly and indirectly, by playing a crucial role in human well-being and societal development [34]. Ecosystem services encompass synergies, with simultaneous increases in multiple services, as well as trade-offs, where increases in one service occur at the expense of another [35,36]. Current research in orchard ecosystems primarily focuses on a limited range of ecosystem services. Comprehensive studies that evaluate multiple ecosystem services concurrently are rare, with those that evaluate the trade-offs and synergies among these services being even scarcer [37]. Identifying how a specific agronomic practice integrated into orchard ecosystems influences various ecosystem services and their interrelations will facilitate the development of innovative orchard ecosystem models and support the needs of various stakeholders while promoting sustainability [33].

In this study, we intercropped flowering plants in an apple orchard to explore the effects of functionally different intercropped plants on various ecosystem services and their interrelations. Our objectives were to (1) evaluate the effects of intercropping flowering plants in an apple orchard on diverse ecosystem service indicators; (2) identify the trade-offs or synergistic relationships among ecosystem services; and (3) conduct a comprehensive evaluation of intercropping flowering plants in apple orchards.

## 2. Materials and Methods

### 2.1. Study Site and Experimental Design

The experiment was conducted in an apple orchard at the Yantai Experimental Station of the Chinese Academy of Sciences, Shandong Province, China (37°43′31″ N, 120°55′17″ E), from the end of April to October in 2020 and 2021. The area has a warm temperate continental monsoon climate, with a mean annual precipitation of 664 mm and a mean annual temperature of 11.7 °C. The orchard soil is predominantly sandy loam. The primary apple variety was ‘Fuji 2001’, and trees were planted in 2014 with a spacing of 4 m × 6 m.

The experiment followed a randomized block design, with four replicate blocks (*n* = 4), each containing one control and four intercropping treatment plots. There were four intercropping flowering plant treatments: *B. napus*, *V. villosa*, *C. monnieri*, and *S. tenuifolia*, which were intercropped between the rows of apple trees. These four flowering plant species were further selected from a local functional-plant pool based on our previous screening and application studies in apple orchards in the Jiaodong Peninsula [38]. Control plots were subjected to clean tillage. Weeds were manually removed throughout the study to ensure that the control plots remained weed free. Each plot was 18 m × 16 m, equivalent to three rows of trees with five trees per row (in the plot). There were 12 m (east–west) and 16 m (north–south) buffer zones separating adjacent plots. Standard agronomic practices of pruning, irrigation, and fertilizing were applied consistently across all orchard plots. After flowering and natural senescence, the flowering plants were mown and retained on the orchard floor as surface mulch/green manure, where they decomposed naturally. No plant residues were removed from the intercropped plots, and no additional external fertilizer was introduced through this residue-management practice.

### 2.2. Assessment of Multiple Ecosystem Service Indicators

**Figure 1:**
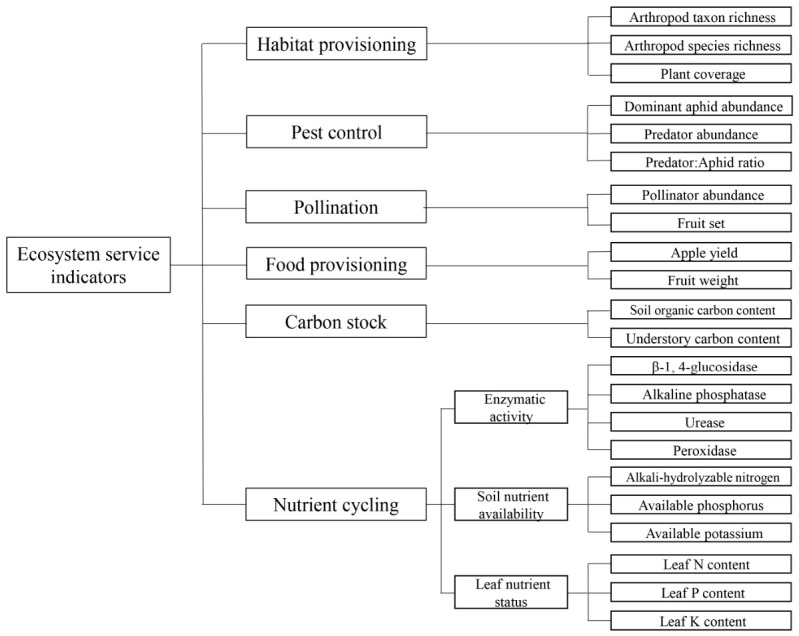
Ecosystem service indices of intercropped flowering plants in an apple orchard. Twenty-two individual ecosystem service indices (**right**) were grouped into six composite ecosystem services indices (**middle**).

Twenty-two ecosystem service indicators were measured in each plot from the end of April to October in 2020 and 2021 (Figure 1). The ecosystem service indicators were classified into six groups: nutrient cycling, habitat provisioning, carbon stock, pollination, pest control, and food provisioning. The classification was largely based on the Common International Classification of Ecosystem Services (CICES) developed by the European Environmental Agency and was adapted with reference to previous ecosystem service assessment approaches [39,40]. Habitat provisioning services included arthropod abundance, arthropod taxon richness, and understory plant cover. Pest control services were primarily indicated by dominant aphid abundance, predator abundance, and the predator-to-aphid ratio. Pollination services include factors such as pollinator abundance and fruit set. Food provisioning services are mainly based on factors such as apple yield and individual fruit weight. Carbon stock services are predominantly assessed by soil organic carbon content and understory carbon content. Nutrient cycling services are primarily indicated by soil enzymatic activity, soil nutrient availability, and leaf nutrient status, with a focus on the specific subdivisions of the nitrogen, phosphorus, and potassium cycles. The overall ecosystem service index was calculated as the sum of the six composite indicators of ecosystem services.

### 2.3. Sampling of Apple Trees for Dominant Aphids and Predators

To determine the population densities of pests and predators on apple trees, trees were visually inspected during early June of 2020 and 2021. Based on our previous work in apple orchards, *Aphis spiraecola* Patch (Hemiptera: Aphididae) was considered the dominant pest, and the main predators included ladybeetles, lacewings, hoverflies, and spiders [21]. Six apple trees in the middle of each plot were sampled. On each tree, one current-year shoot was sampled from each of the four cardinal directions (east, south, west, north). The number and species of predators in the distal 50 cm of each branch and the number of pests in the distal 10 cm of each branch including leaves and shoots were counted. All visible *A. spiraecola* individuals, including nymphs and adults, present on leaves and tender shoots within the distal 10 cm of each sampled branch were directly counted in the field. No subsampling or population-scaling approach was applied. All visible predator individuals within the distal 50 cm of each sampled shoot were directly counted in the field. For ladybeetles, both larvae and adults were counted as predatory stages. For lacewings and hoverflies, only larvae observed on apple shoots were included as predatory natural enemies, because lacewing and hoverfly adults primarily feed on floral resources such as nectar, pollen, or honeydew. For spiders, both juvenile and adult individuals were counted. Eggs, pupae, empty exuviae, and cocoons were not included in predator abundance. No subsampling or population-scaling approach was applied for predator counts. Thus, 24 shoots were surveyed per plot, and counts were summarized to obtain plot-level values for dominant aphid abundance and predator abundance. Aphid abundance was expressed as the mean number of *A. spiraecola* individuals per 10 cm shoot at the plot level. Predator abundance was expressed as the mean number of predator individuals per 50 cm shoot at the plot level.

### 2.4. Sampling of Intercropped Flowering Plants for Arthropods

Arthropods were sampled using yellow sticky traps during early June in 2020 and 2021. Arthropods captured by yellow sticky traps were identified to the lowest possible taxonomic level and were used to calculate arthropod abundance and taxon richness. Each treatment plot, including the clean-tillage control plot, was subdivided equally into three subplots. Yellow sticky traps (25 cm × 20 cm) were suspended in each subplot. In flowering plant plots, trap height was adjusted as plants grew to maintain the bottom of the yellow card approximately 10 cm above the plant canopy. In clean-tillage control plots without understory vegetation, traps were placed at the same relative subplot positions and at a standardized height above the orchard floor. Traps were installed three days before the survey to effectively determine the diversity and abundance of arthropod taxa in each subplot. Thus, three yellow sticky traps were used per plot and exposed for 72 h; arthropod abundance and taxon richness were calculated at the plot level. Identification of captured arthropods was based mainly on external morphological characters using published taxonomic keys and reference works for Chinese insects and common orchard arthropods, including an illustrated guide to insects from Wangjiayuan, Beijing [41], a photographic guide to Chinese insects [42], the taxonomic reference for Chinese bees [43], and the taxonomic reference for Chinese Syrphidae [44]. When specimens could not be reliably identified to species level, they were retained at the genus, family, order, or morphotaxon level and used for taxon-richness calculations.

### 2.5. Sampling of Pollinators

Pollinators were sampled using pan traps during the apple blooming period in 2020 and 2021. Pan traps are an effective and standardized tool for sampling flower-visiting insects and potential pollinators, while minimizing observer bias [45]. Three color plates (blue, yellow, and white) were positioned at the center of each plot, filled with 200 mL of a soapy water solution at a concentration of approximately 4.0 g/L, and spaced 2–3 m apart. Pan traps were set up at 9:00 AM in all plots and removed at 4:00 PM. Trapped arthropods were preserved in 70% ethanol and grouped according to pan trap color. Specimens were subsequently stored at −18 °C for future identification. Individuals were identified based on morphological characteristics to the subfamily or family level or, when possible, to a lower taxonomic level. Bees were identified mainly based on external morphological characters using the taxonomic reference for Chinese bees [43], and syrphid flies were identified using the taxonomic reference for Chinese Syrphidae [44]. Previous studies on apple flower-visiting insects were also used to assist ecological grouping of pollinators [46,47]. When specimens could not be reliably identified to species level, they were retained at the genus, family, order, or morphotaxon level and used for taxon-richness calculations. Subsequently, based on previous studies of apple pollinators, two major pollinator groups were considered in this study: flower-visiting Hymenoptera, particularly Apoidea such as Apidae, Andrenidae, and Megachilidae, and flower-visiting Diptera, especially Syrphidae [48,49]. The three pan traps within each plot were pooled to calculate plot-level captured potential pollinator abundance. The main pest, natural-enemy, and potential pollinator taxa recorded in the orchard, together with their sampling methods and corresponding response variables, are summarized in Table 1. Complete annual taxonomic inventories are provided in Supplementary Tables S6 and S7. Treatment-specific mean abundances (±SE) of the most abundant pest, natural-enemy, and pollinator taxa are provided in Supplementary Table S8.

### 2.6. Fruit Set Rate

Fruit set rates were investigated in the apple orchard during the peak flowering season in 2020 and 2021. Six apple trees were selected in each plot, and then, one branch was chosen from each of the four directions (east, south, west, north), and the number of flowers on each tree was counted. The number of fruits on the selected branches was counted after the natural fruit drop period (about 25 d later). The fruit set rate was then calculated as the number of fruits divided by the number of flowers.

### 2.7. Yield of Apple Trees

Apple yields were measured during the apple maturation period in late October in 2020 and 2021. Three apple trees were selected in each plot, and each tree was individually harvested and the fruits weighed. The number of fruits per tree was recorded, and the weight of each fruit was measured using an electronic scale (Shanghai Puchun Measuring Instruments Co., Ltd., Songjiang, China).

### 2.8. Aboveground Biomass and Carbon Content

Aboveground biomass and carbon content were sampled in mid-June in 2020 and 2021. At each sampling point, three subplots were sampled using a 50 cm × 50 cm quadrat, serving as replicates for horizontal comparison. All aboveground vegetation in the subplots was cut manually and then oven-dried at 80 °C for 24 h in the laboratory to determine the dry weight. The carbon content was determined by multiplying the dry biomass by the carbon fraction factor of 0.47. This value represented the standing aboveground carbon content of the understory vegetation at the time of sampling and was used as a relative carbon-related indicator, rather than as an absolute estimate of total orchard carbon storage.

### 2.9. Vegetation Coverage

Plant coverage was estimated in mid-June of 2020 and 2021. In each treatment plot, three points were sampled using a 1 m × 1 m quadrat, serving as replicates for horizontal comparison. Plant coverage was determined using grid-based visual estimation.

### 2.10. Nutritional Status of Apple Leaves

Apple leaf samples were collected in early July of 2020 and 2021. Six apple trees in the middle of each plot were sampled to determine apple leaf nutritional status. Five mature leaves were removed from new shoots from four directions (east, south, west, north) of each tree, totaling 120 leaves per treatment plot. In the laboratory, leaves were washed with distilled water and initially heated at 105 °C for 15–30 min to halt any biological activity. Leaves were then oven-dried at 80 °C to a constant weight, ground, and sieved. Samples were digested using H_2_SO_4_–H_2_O_2_. Total nitrogen was determined using a Kjeldahl method, total phosphorus content by a molybdenum blue colorimetric method, and total potassium content by flame photometry.

### 2.11. Soil Sampling

Soils were sampled in early September of 2020 and 2021. Soils (0–20 cm) were sampled using the five-point sampling method in each plot, for a total of five soil samples per plot, which were thoroughly mixed to form a single composite sample. Soil samples were passed through a 2 mm sieve and then divided into two parts. One part was stored at −18 °C in a freezer for determination of soil enzyme activity, while the other part was air-dried for determination of soil properties.

Soil organic matter was determined using the H_2_SO_4_–K_2_Cr_2_O_7_ wet-oxidation. Soil organic carbon (SOC) content was calculated by multiplying the soil organic matter content by 0.5. Alkali-hydrolyzable nitrogen (AN) was determined using an alkali hydrolysis reduction diffusion method, available phosphorus (AP) by colorimetry, and available potassium (AK) by flame photometry [50].

Activity was determined for the following enzymes: β-1, 4-glucosidase, alkaline phosphatase, urease, and peroxidase. β-1,4-glucosidase is used as a measure of cellulose decomposition, alkaline phosphatase as a measure of phosphorus cycling, urease as a measure of nitrogen cycling (urease), and peroxidase as a measure of microbial activity. Soil enzyme activity was determined using a microplate fluorescence method [51].

### 2.12. Statistical Analyses

#### 2.12.1. Individual Ecosystem Service Indices

All statistical analyses were performed using R software (version 4.2.0; R Core Team, Vienna, Austria). To assess the effect of intercropping flowering plants on each ecosystem service indicator, generalized linear mixed models (GLMMs) from the “glmmTMB” package (version 1.1.14) were used for hypothesis testing. Ecosystem service indicators were used as response variables, with treatment (four flowering plants or untreated control) as the fixed factor. Block groups were treated as random factors. An interaction between treatment and block group was not included because of the small sample size. We conducted model selection with likelihood-ratio tests and retained the minimum adequate models for subsequent statistical inference. Chi-square statistics were derived from these likelihood-ratio comparisons (Supplementary Table S1). The “Anova” function in the “car” package (version 3.1-2) was employed to determine the significance of fixed effects in these models, with Tukey’s pairwise comparisons using the “glht” function in the “multcomp” package (version 1.4-26) applied to models that showed significant effects. Effects were considered significant at *p* < 0.05. The plot was used as the experimental unit for all arthropod-related analyses.

#### 2.12.2. Composite Indices

To better compare and visualize the effects of various treatments on multiple ecosystem services, we developed composite indices from groups of ecosystem services following an approach similar to that proposed by Vincent et al. [40]. The values of individual ecosystem service indicators were converted into a common scoring unit ranging from 0.1 to 1, using the following homothetic transformations:

(1) $$Y_{i}=0.1+\left(\frac{x_{i}-min_{i}}{max_{i}-min_{i}}\right)\times 0.9$$

(2) $$Y_{i}=1.1-\left(0.1+\left(\frac{x_{i}-min_{i}}{max_{i}-min_{i}}\right)\times 0.9\right)$$

where *i* represents the ecosystem service indicator index, *Y* is the response index value of *i*, and *max* and *min* represent the maximum and minimum of *i*, respectively. For the variables on a “more-is-better” scale, e.g., natural enemy abundance, Equation (1) was used for the transformation, whereas for those on a “more-is-worse” scale, e.g., dominant aphid abundance, Equation (2) was used for the reverse transformation.

The value of each composite index of an ecosystem service group was calculated as the weighted sum of all transformed variables within each group. Based on principal component analysis (PCA), weights were applied according to the relative contribution of each variable to the variance within the group of ecosystem service composite indicators. The factor scores of each variable on the initial two axes were used as weights, with the final index calculated as follows:

(3) $$CI=\sum_{i=0}^{n}\left(Y_{i}w_{i,PC1}+Y_{i}w_{i,PC2}\right)$$

where *CI* represents the composite index, *Y_i_* is the transformed value of each variable *i* according to Equation (1) or (2), and the *w* are the factor scores derived from the first and second principal component axes. Last, each composite index was again transformed to the range of 0.1–1 according to Equation (1).

#### 2.12.3. Principal Component Analysis of Orchard Ecosystem Service Composite Indices

Principal component analysis was applied to assess the trade-offs and synergistic effects among the six ecosystem service composite indices. A synergy between two or more ecosystem services is indicated when they have high positive loadings on the same principal component (PC). Trade-offs are indicated when one ecosystem service aligns positively on a PC axis, while another aligns negatively. To select which PC axes were relevant, the contribution of each axis to the explained variance was used, and then, the minimum number of axes explaining more than 70% of the variance was selected.

## 3. Results

### 3.1. Individual Ecosystem Service Indicators

#### 3.1.1. Habitat Provision and Pest Control Services

The pest control analysis was based primarily on the abundance of the dominant aphid, *Aphis spiraecola*, observed on apple shoots. Therefore, treatment effects on this indicator should be interpreted as changes in dominant aphid abundance rather than as evidence that all minor pest taxa differed significantly from the control. Arthropod abundance, arthropod taxon richness, and plant cover were the three ecosystem service indicators associated with the composite habitat provisioning index. (Supplementary Tables S2 and S3). In 2020, arthropod abundance in the *C. monnieri* and *B. napus* plots was significantly higher than that in control plots (χ^2^ = 6.276; *df* = 4; *p* < 0.001). In 2021, arthropod abundance in the *C. monnieri*, *V. villosa*, and *B. napus* plots was significantly higher than that in control plots (χ^2^ = 31.262; *df* = 4; *p* < 0.001). Compared with other intercropped treatments, arthropod abundance was highest in the *C. monnieri* plots in 2020 and 2021.

In 2020, arthropod species richness in the *C. monnieri* and *B. napus* plots was significantly higher than that in control plots (χ^2^ = 24.338; *df* = 4; *p* < 0.001). In 2021, arthropod richness in the four flowering plant plots was significantly higher than that in control plots (χ^2^ = 24.338; *df* = 4; *p* < 0.001). Compared with other intercropped treatments, arthropod richness was highest in the *C. monnieri* plots in 2020 and 2021. Plant coverage in the control plots was zero because of effective manual weeding, but it was significantly higher in the *C. monnieri* and *V. villosa* plots than in those of the other treatment in 2020 and 2021.

Dominant aphid abundance, predator abundance, and the predator-to-aphid ratio were the three ecosystem service indicators associated with the composite pest control index. Pest abundance in the *C. monnieri* and *V. villosa* plots was significantly lower than that in control plots in 2020 and 2021 (2020: χ^2^ = 17.124; *df* = 4; *p* < 0.001; 2021: χ^2^ = 43.761; *df* = 4; *p* < 0.001). Compared with other intercropped treatments, pest abundance was lowest in the *C. monnieri* plots in 2020 and 2021.

Predator in the *C. monnieri* plots was significantly higher than that in control plots in 2020 and 2021 (2020: χ^2^ = 100.27; *df* = 4; *p* < 0.001; 2021: χ^2^ = 10.197; *df* = 4; *p* < 0.001). The enemy-to-pest ratio in the *C. monnieri* plots was significantly higher than that in control plots in 2020 (χ^2^ = 24.148; *df* = 4; *p* < 0.001), and in 2021 the enemy-to-pest ratio in the *C. monnieri* plots was significantly higher than that in other treatment plots (χ^2^ = 59.085; *df* = 4; *p* < 0.001).

#### 3.1.2. Pollination, Food Provisioning, and Carbon Stock Services

Pollinator abundance and fruit set were the two ecosystem service indicators associated with the composite pollination index (Supplementary Tables S2 and S3). In 2020, pollinator abundance in the *B. napus* and *V. villosa* plots was significantly higher than that in control plots (χ^2^ = 15.542; *df* = 4; *p* = 0.0037). In 2021, pollinator abundance in the *B. napus* plots was significantly higher than that in control and *S. tenuifolia* plots (χ^2^ = 31.262; *df* = 4; *p* < 0.001). In 2020, fruit set in the *B. napus* and *V. villosa* plots was significantly higher than that in control plots (χ^2^ = 23.991; *df* = 4; *p* < 0.001). In 2021, fruit set in the *B. napus*, *V. villosa*, and *C. monnieri* plots was significantly higher than that in control plots (χ^2^ = 15.21; *df* = 4; *p* = 0.0043).

Apple yield and fruit weight were the two indicators associated with the composite food provisioning index (Supplementary Tables S2 and S3). In 2020, apple yield in the *V. villosa* plots was significantly higher than that in control plots (χ^2^ = 17.412; *df* = 4; *p* = 0.0016). In 2021, apple yield in the *C. monnieri* plots was significantly higher than that in control and *S. tenuifolia* plots (χ^2^ = 20.616; *df* = 4; *p* < 0.001). There were no differences in individual fruit weight among treatments and control in 2020 and 2021. However, the highest individual fruit weight was in *V. villosa* plots in both years.

Soil organic carbon content and understory carbon content were the two ecosystem service indicators associated with the composite carbon stock index (Supplementary Tables S2 and S3). In 2020, soil organic carbon content in the *B. napus* plots was significantly higher than that in control plots (χ^2^ = 11.981; *df* = 4; *p* = 0.0175). In 2021, soil organic carbon content was significantly higher in the four flowering plant plots than in control plots (χ^2^ = 27.698; *df* = 4; *p* < 0.001). The understory carbon content in the control plots was assumed to be zero, because manual weeding kept the ground bare. The understory carbon content in the *C. monnieri* plots was significantly higher than that in the *V. villosa* and *S. tenuifolia* plots in both years (2020: χ^2^ = 268.06; *df* = 4; *p* < 0.001; 2021: χ^2^ = 219.64; *df* = 4; *p* < 0.001).

#### 3.1.3. Nutrient Cycling Services

Enzymatic activity, soil nutrient availability, and leaf nutrient status were the three ecosystem service indicators associated with the composite nutrient cycling index (Supplementary Tables S2 and S3). The above three ecosystem service indicators are closely related to the cycling of nitrogen, phosphorus, and potassium.

In this study, we selected enzymes based on their collective ability to offer a comprehensive overview of soil decomposition processes and activities in key biogeochemical cycles. The activity of β-glucosidase in the four flowering plant plots was significantly higher than that in control plots in 2020 and 2021 (2020: χ^2^ = 30.463; *df* = 4; *p* < 0.001; 2021: χ^2^ = 71.95; *df* = 4; *p* < 0.001). Alkaline phosphatase activity in the *V. villosa* plots was significantly higher than that in control plots in 2020 (χ^2^ = 30.463; *df* = 4; *p* < 0.001); In 2021, alkaline phosphatase activity in the four flowering plant plots was significantly higher than that in control plots (χ^2^ = 131.32; *df* = 4; *p* < 0.001); and alkaline phosphatase in the *V. villosa* plots was highest compared with that in other treatment plots. Urease activity in *C. monnieri*, *V. villosa*, and *S. tenuifolia* plots was significantly higher than that in control plots in 2020 (χ^2^ = 23.731; *df* = 4; *p* < 0.001). In 2021, urease activity in the *B. napus* plots was significantly higher than that in control plots (χ^2^ = 10.252; *df* = 4; *p* = 0.0364). Peroxidase activity in the *C. monnieri* plots was significantly higher than that in control plots in 2020 (χ^2^ = 70.995; *df* = 4; *p* < 0.001). In 2021, peroxidase activity in the *B. napus* and *V. villosa* plots was significantly higher than that in control plots (χ^2^ = 41.878; *df* = 4; *p* < 0.001).

To assess nutrient availability, contents of alkali-hydrolyzable nitrogen, available phosphorus, and available potassium were measured. Alkali-hydrolyzable nitrogen in the *V. villosa* and *B. napus* plots was significantly higher than that in control plots in 2020 (χ^2^ = 18.791; *df* = 4; *p* < 0.001). In 2021, alkali-hydrolyzable nitrogen in the *V. villosa* plots was significantly higher than that in control plots (χ^2^ = 11.423; *df* = 4; *p* = 0.0222). Available phosphorus in the *B. napus* and *C. monnieri* plots was significantly higher than that in control plots in 2020 and 2021 (2020: χ^2^ = 20.485; *df* = 4; *p* < 0.001; 2021: χ^2^ = 13.72; *df* = 4; *p* = 0.0082). Available potassium in the *V. villosa*, *C. monnieri*, and *S. tenuifolia* plots was significantly higher than that in control plots in 2020 (χ^2^ = 17.197; *df* = 4; *p* = 0.0018). In 2021, available potassium was not affected by intercropping treatments.

Leaf nutrient status was determined by measuring leaf nitrogen, phosphorus, and potassium contents. There were no significant differences in leaf nitrogen and phosphorus contents among intercropped treatment plots in 2020 and 2021. Potassium content of leaves in the *B. napus* and *C. monnieri* plots was significantly higher than that in control plots in 2020 (χ^2^ = 15.066; *df* = 4; *p* = 0.0046). In 2021, leaf potassium content in the *C. monnieri* plots was significantly higher than that in control plots (χ^2^ =10.925; *df* = 4; *p* = 0.0274).

### 3.2. Composite Ecosystem Service Indices

**Figure 2:**
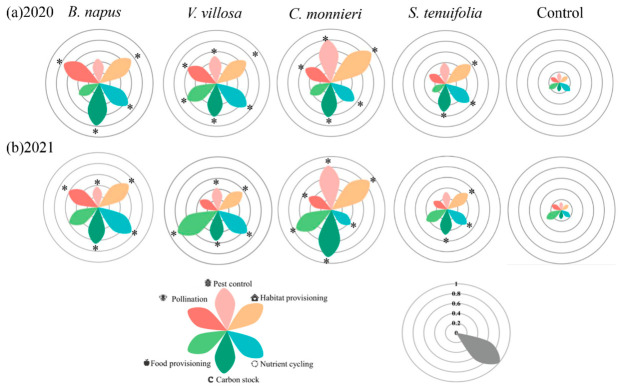
Flower diagrams of mean scores of six composite ecosystem service indices in the flowering plants and control plots in (**a**) 2020 and (**b**) 2021. Composite ecosystem service indices are identified by color: red for pollination, pink for pest control, orange for habitat provisioning, blue for nutrient cycling, dark green for carbon stock, and light green for food provisioning. Petal lengths of the flowers reflect transformed effect sizes based on Equations (1) and (2), with the circles representing the value of each ecosystem service index on a scale of 0 to 1. An asterisk (*) indicates a significant difference between an intercropped flowering-plant treatment and the untreated control at *p* < 0.05.

The scores for the six composite nutrient cycling, carbon stock, habitat provisioning, pest control, pollination, and food provisioning ecosystem service indices were determined in the flowering plants and control plots in 2020 and 2021 (Figure 2 and Supplementary Tables S4 and S5). The scores of the composite index for pest control in the *C. monnieri* and *V. villosa* plots were significantly higher than those in the control plots in 2020 (*F* = 6.276; *df* = 4; *p* < 0.001); Similarly, in 2021, the scores of the composite pest control index in the *C. monnieri*, *V. villosa*, and *B. napus* plots were significantly higher than those in the control plots (*F* = 18.92; *df* = 4; *p* < 0.001). The score of the composite index for food provisioning in the *V. villosa* plots was significantly higher than that in the control plots in 2020 (*F* = 3.407; *df* = 4; *p* = 0.0359). The result was similar in 2021 with a significantly higher food provisioning composite index in *C. monnieri* and *V. villosa* plots than in the control plots (*F* = 3.21; *df* = 4; *p* = 0.0432). In 2020, the composite pollination index in the *V. villosa*, *B. napus*, and *C. monnieri* plots was significantly higher than that in the control plots (*F* = 4.561; *df* = 4; *p* = 0.0131). Results were similar in 2021, with the composite pollination index significantly higher in *B. napus* and *C. monnieri* plots than that in the control plots (*F* = 3.576; *df* = 4; *p* = 0.0307). In both 2020 and 2021, the composite carbon stock index in the four flowering plant plots was significantly greater than that in the control plots (2020: *F* = 6.139, *df* = 4, *p* = 0.0039; 2021: *F* = 7.757, *df* = 4, *p* = 0.0014).

The composite habitat provisioning index in *C. monnieri* plots was significantly higher than that in other plots in both 2020 and 2021 (2020: *F* = 26.7; *df* = 4; *p* < 0.001; 2021: *F* = 42.78; *df* = 4; *p* < 0.001). The composite nutrient cycling index in the four flowering plant plots was significantly greater than that in the control plots in both years (2020: *F* = 4.044; *df* = 4; *p* = 0.0202; 2021: *F* = 19.96; *df* = 4; *p* < 0.001).

**Figure 3:**
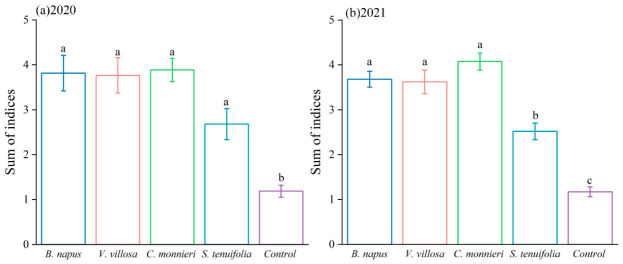
Total ecosystem services index per intercropped flowering plant treatment in (**a**) 2020 and (**b**) 2021. The total index was calculated as the sum of six composite ecosystem services indices. Data are the mean ± SE. Different lowercase letters indicate a significant difference among treatments at *p* < 0.05, according to one-way ANOVA with Tukey’s test.

The scores of overall ecosystem services in the four flowering plant plots were significantly higher than those in the control plots in 2020 and 2021 (2020: *F* = 13.12; *df* = 4; *p* = 0.0202; 2021: *F* = 38.38; *df* = 4; *p* < 0.001) (Figure 3). Compared with control plots, the mean increase in the overall ecosystem service score was 228% in *C. monnieri* plots, 222% in *B. napus* plots, 217% in *V. villosa* plots, and 126% in *S. tenuifolia* plots in 2020, and in 2021, the increase was 248%, 214%, 209%, and 115%, respectively.

### 3.3. Ecosystem Services Synergies and Trade-Offs

**Figure 4:**
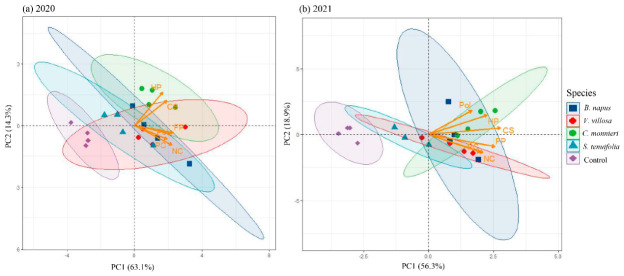
Biplot of the first two principal components (PC) of all composite ecosystem service indices for each intercropped flowering plant treatment in (**a**) 2020 and (**b**) 2021. The percentages shown on each axis indicate the proportion of total variation explained by the first two PCs. The angle between arrows indicates correlation between individual variables, with correlations increasing as the angle decreases. NC = nutrient cycling, HP = habitat provisioning, CS = carbon stock, Pol = pollination, PC = pest control, and FP = food provisioning.

We reduced the six-dimensional space to two dimensions, in which the selected components (PC1, PC2) had eigenvalues higher than 1 and accounted for more than 70% of the total variance cumulatively (Figure 4). In 2020, the selected components (PC1, PC2) had eigenvalues higher than 1 and accounted for 77.40% of the total variance. The contribution of the first principal component to the variance was 63.10%, with the main contributing indicators being food provisioning and nutrient cycling. The second principal component accounted for 14.30% of the variance, with the main contributing indicators being carbon stock and habitat provisioning. In 2021, the first two principal components explained 75.20% of the total variance. The first principal component accounted for 56.30% of the variance, mainly driven by the indicators of food provisioning and nutrient cycling; the second principal component accounted for 18.90% of the variance, mainly influenced by the indicators of habitat provisioning and carbon sequestration.

According to the direction of the arrows in 2020 (Figure 4a) along the *x* axis, all six composite ecosystem service indicators were on the positive half of the *x* axis, suggesting potential synergistic effects among the composite indicators. Along the *y* axis, the indicators for habitat provisioning and carbon stock were positively correlated, as were the indicators for food provisioning, nutrient cycling, and pest control. In 2021 (Figure 4b), along the *x* axis, all six composite ecosystem service indicators were on the positive half of the axis, indicating potential synergistic effects among the indicators. Along the *y* axis, the indicators for habitat provisioning and carbon stock were positively correlated, as were the indicators for food provisioning, nutrient cycling, and pest control. In 2020, the points representing the control plots in the diagram were well separated from those of the four flowering plant plots; a similar pattern was observed in 2021, with the points of the control plots distinctly separated from those of the four flowering plant plots. These results suggest that the measured composite ecosystem service indicators differed between flowering plant plots and clean-tillage control plots.

## 4. Discussion

In this study, flowering plants were intercropped between apple tree rows to form a diversified orchard-floor management system. Twenty-two ecosystem service indicators were chosen to establish a framework for assessing ecosystem services in apple orchards. The ecosystem service indicators were grouped into six composite indicators: pollination, pest control, habitat provisioning, nutrient cycling, carbon stock, and food provisioning. There were significant differences in the six composite indicators between the four flowering plant plots and control plots. The overall index of ecosystem services in the four flowering plant plots was significantly greater than that in the control plots. These results show that intercropping flowering plants supports multiple ecosystem services in apple orchards.

Aboveground, intercropped flowering plants supply beneficial arthropods with pollen, nectar, and alternative prey, while also furnishing oviposition sites, refuges, and sheltered microhabitats for overwintering and summer aestivation. As a result, populations of natural enemies and pollinating insects increase, and flowering plants become sites for the breeding of beneficial insects in the field [21,52,53]. By attracting natural enemies with flowering plants, a top-down approach that leverages the plants–pests–predators food chain can be an effective strategy for controlling pest populations [54]. In a previous study, we found that four flowering plants conserve different numbers and proportions of the main predator species, including ladybeetles, lacewings, hoverflies, and spiders, and those natural enemies were able to transfer to fruit trees and improve control of pests such as the apple aphid [21]. In similar research, *Lobularia maritima* L. and *Sinapis alba* L. (Brassicales: Brassicaceae) planted around peach orchards increased the number of hoverflies and parasitic wasps on adjacent peach trees, thereby increasing biological control of peach aphids [55]. From the perspective of pollination ecosystem services, apple flowers are highly reliant on insect pollination for successful fruit set because of the hermaphroditic and self-incompatible nature of apple [56,57,58]. In this study, the flowering period of *B. napus* was slightly earlier than but overlapping with that of apple, which facilitated the early introduction and conservation of pollinating insects and increased the fruit set rate of apple flowers. Intercropping flowering plants helps to sustain a wide range of pollinator species and increases the number of pollinators, leading to improved pollination and increased fruit set in apple orchards [59,60].

Belowground, intercropping flowering plants in orchards can increase soil organic matter, stimulate soil enzyme activities, and improve soil fertility [17,27]. Flowering plants may potentially compete with fruit trees for soil nutrients and water, particularly under resource-limited conditions or inappropriate orchard-floor management [17,38]. However, in the present study, leaf N and P contents were not significantly reduced in the intercropped plots, individual fruit weight was not reduced, and apple yield was maintained or increased in some flowering plant treatments, suggesting that strong nutrient competition was not evident under our experimental conditions. For example, leguminous plants can fix significant amounts of nitrogen, thereby enriching soil nitrogen levels. Moreover, their low carbon-to-nitrogen ratio leads to rapid decomposition, ultimately boosting soil fertility [61]. In this study, the legume *V. villosa* increased soil alkali-hydrolyzable nitrogen and urease activity, which could increase overall soil fertility. Soil enzymes, secreted as extracellular enzymes by soil bacteria and fungi, are crucial in breaking down complex organic compounds and making nutrients available for plant uptake [62,63]. In this study, the levels of the four soil enzymes in the four flowering plant plots were higher than those in control plots, possibly because of increases in the number of soil microorganisms under intercropping with flowering plants. Intercropping with the flowering plants *Ageratum houstonianum* Mill. and *Tagetes patula* L. in apple orchards leads to increases in the activities of transformative soil enzymes and nitrogen-fixing enzymes and thus promotes carbon–nitrogen cycling and improves soil structure [64]. Because the same fertilization regime was applied across all treatments, this improvement was more likely associated with enhanced internal nutrient cycling and nutrient availability rather than increased external fertilizer input [17,27].

The diversification of plant species or varieties in agroecosystems can increase multiple ecosystem services such as pollination, pest control, nutrient cycling, soil fertility, and moisture regulation, without negatively affecting crop yields [33]. In this study, there were possible synergies among the six composite ecosystem service indices, but no trade-offs. Implementing tailored flower strips is highly effective, providing multiple functions, such as supporting biodiversity, reducing pests, and improving crop yields, which can significantly contribute to ecological intensification in agriculture [65]. Similarly, inter-row grass growth in apricot orchards increases various ecosystem services while maintaining or increasing the level of food supply [40]. Careful selection of flowering plant species is essential based on experimental objectives, because different flowering plants have different promotional effects on ecosystem services under different times, conditions, and environments [66]. Our results clearly demonstrate the importance of selecting different intercropping flowering plants depending on the ecosystem service that needs to be improved, as demonstrated by the relatively high pest control services of *C. monnieri*, the beneficial pollination services of *B. napus*, and the increase in soil fertility with *V. villosa*.

This study has several limitations regarding the insect-related indicators. First, arthropod richness was evaluated as taxon richness rather than strict species richness, because not all specimens could be reliably identified to species level. Second, pollinator abundance was estimated using pan traps during apple bloom, which provides a standardized indicator of captured potential pollinator abundance but does not directly quantify flower visitation rate or pollination efficiency. In addition, pan traps and yellow sticky traps mainly measure the abundance of captured insects rather than directly quantifying actual plant or floral visitation behavior because some captured individuals may only pass through the sampling area. Moreover, these trapping methods may unintentionally remove beneficial arthropods during sampling. Third, pest control was evaluated using dominant aphid abundance, predator abundance, and the predator-to-aphid ratio, rather than direct exclusion experiments or measurements of predation rate. Therefore, our conclusions are restricted to the measured ecosystem service indicators under the conditions of this two-year orchard experiment. Future studies should combine trapping methods with direct observations, such as visual counts of pollinators or beating (frappage) methods, to better characterize plant–insect interactions.

In summary, intercropping with flowering plants increased various ecosystem services within the apple orchard. We developed a framework to measure multiple ecosystem service indicators in orchard environments by selecting twenty-two ecosystem service indicators and grouping them into composite indices of nutrient cycling, habitat provisioning, carbon stock, pollination, pest control, and food provisioning. The overall ecosystem services index in plots with the four flowering plant species was significantly greater than that in control plots. These findings indicate that intercropping flowering plants can serve as a promising orchard-floor management strategy to improve the ecological functioning and sustainability of apple production systems. Based on the results of this two-year experiment, *C. monnieri* appears to be the most suitable single species for improving overall orchard multifunctionality under the conditions of this study, especially because of its strong performance in habitat provisioning and pest control. When specific management goals are considered, *B. napus* may be preferred for enhancing pollination services, whereas *V. villosa* may be more suitable for improving soil fertility and nutrient cycling. Future research should incorporate a wider spatial and temporal perspective, as well as a more comprehensive context, to ensure that conservation biological control methods, such as intercropping flowering plants in orchards, are both ecologically sustainable and economically viable, while also meeting social acceptance criteria.

## Figures and Tables

**Table 1:** Main pest, natural-enemy, and potential pollinator taxa recorded in the apple orchard, together with their sampling methods and corresponding response variables.

Ecological Component	Main Taxon or Group	Representative Taxa Recorded in the Orchard	Sampling Method	Response Variable
Dominant apple pest	Aphididae	*Aphis spiraecola* Patch (Hemiptera: Aphididae)	Visual inspection of apple shoots	Dominant aphid abundance
Predatory ladybeetles	Coccinellidae	*Harmonia axyridis*, *Propylea japonica*, and *Hippodamia variegata*	Visual inspection of apple shoots	Predator abundance
Predatory lacewings	Chrysopidae	*Chrysopa pallens* and Chrysopidae morphotaxa	Visual inspection of apple shoots	Predator abundance
Aphidophagous hoverflies	Syrphidae	*Episyrphus balteatus*, *Eupeodes corollae*, and other Syrphidae morphotaxa	Visual inspection of apple shoots	Predator abundance
Predatory spiders	Araneae, including Thomisidae	*Ebrechtella tricuspidata* and unidentified Araneae morphotaxa	Visual inspection of apple shoots	Predator abundance
Potential pollinating bees	Apoidea	*Apis mellifera* and other bee taxa identified to the lowest reliable taxonomic level	Pan traps during apple bloom	Captured potential pollinator abundance
Potential pollinating hoverflies	Syrphidae	*E. balteatus*, *E. corollae*, and other Syrphidae morphotaxa	Pan traps during apple bloom	Captured potential pollinator abundance

## Data Availability

The raw data supporting the conclusions of this article will be made available by the authors on request.

## Supplementary Materials

The following supporting information can be downloaded at: https://www.mdpi.com/article/10.3390/plants15142181/s1.
