# Supplementary material for: Intercropping Flowering Plants Enhances Multiple Ecosystem Services in Apple Orchards
Source: Plants (Basel). 2026 Jul 16;15(14):2181. doi: 10.3390/plants15142181 (PMC13414753; doi:10.3390/plants15142181)
Supplement: Supplementary file 1 [file plants-15-02181-s001.zip › plants-4312351-supplementary.pdf]

Supplementary Contents

Table S1. An overview of the distributions applied to each ecosystem service indicator, as determined by the likelihood ratio test.

Table S2. Generalized linear mixed model (GLMM) results for individual ecosystem service (ES) indicators in each intercropped flowering plant treatment in 2020. Data are the mean ± standard error. Different lowercase letters denote significant differences ($p < 0.05$) among treatments based on Tukey's pairwise comparisons.

Table S3. Generalized linear mixed model (GLMM) results for individual ecosystem service (ES) indicators in each intercropped flowering plant treatment in 2021. Data are the mean ± standard error. Different lowercase letters denote significant differences ($p < 0.05$) among treatments based on Tukey's pairwise comparisons.

Table S4. Results of statistical analysis of six ecosystem service composite indicators by each treatment in 2020. Table includes the mean and standard error (SE) per treatment, P value and F value. Different letters denote significant differences ($p < 0.05$) between treatments based on Tukey pairwise comparisons.

Table S5. Results of statistical analysis of six ecosystem service composite indicators by each treatment in 2021. Table includes the mean and standard error (SE) per treatment, P value and F value. Different letters denote significant differences ($p < 0.05$) between treatments based on Tukey pairwise comparisons.

Table S6. Arthropod taxa recorded in 2020 after taxonomic cleaning, with ecological roles and identification levels.

Table S7. Arthropod taxa recorded in 2021 after taxonomic cleaning, with ecological roles and identification levels.

Table S8. Mean abundance (± SE) of the most abundant insect taxa under different flowering-intercrop treatments in 2020 and 2021. Values are presented as means ± SE based on four replicate plots. Pest and natural-enemy taxa were identified to species level, whereas bees were identified and quantified collectively at the superfamily level as Apoidea. The taxa presented in this table were the most abundant taxa within their respective functional groups across the two study years.

Table S1. An overview of the distributions applied to each ecosystem service indicator, as determined by the likelihood ratio test.

**ES indicator**	**Distribution**
**Habitat provisioning**	
Arthropod abundance	Poisson
Arthropod taxon richness	Gaussian
Plant coverage	Gaussian
**Pest control**	
Dominant aphid abundance	Negative binomial
Predator abundance	Gaussian
Predator:Aphid ratio	Gaussian
**Pollination**	
Pollinator abundance	Negative binomial
Fruit set	Gamma
**Food provisioning**	
Apple yield	Gamma
Fruit weight	Gaussian
**Carbon stock**	
Soil organic carbon content	Gaussian
Understory carbon content	Gaussian
**Nutrient cycling**	
**Enzymatic activity**	
β-1, 4-glucosidase	Gaussian
Alkaline phosphatase	Gaussian
Urease	Gaussian
Peroxidase	Gaussian
**Soil nutrient availability**	
Alkali-hydrolyzable nitrogen	Gaussian
Available phosphorus	Gaussian
Available potassium	Gaussian
**Leaf nutrient status**	
Leaf N content	Gaussian
Leaf P content	Gaussian
Leaf K content	Gaussian

Table S2. Generalized linear mixed model (GLMM) results for individual ecosystem service (ES) indicators in each intercropped flowering plant treatment in 2020. Data are the mean ± standard error. Different lowercase letters denote significant differences ($p < 0.05$) among treatments based on Tukey's pairwise comparisons.

ES indicator	Unit	χ^2	GLMM p-value	*Brassica napus*	*Vicia villosa*	*Cnidium monnieri*	*Schizonepeta tenuifolia*	Control
**Habitat provisioning**								
Arthropod abundance	# of individuals	31.26	< 0.0001 ***	42.92 ± 5.71 ab	40.08 ± 2.8 abc	51.92 ± 1.7 a	36.75 ± 3.66 bc	27.5 ± 5.36 c
Arthropod taxon richness	no. of taxa per plot	24.34	< 0.0001 ***	12.25 ± 1.23 a	11.08 ± 1.77 ab	14.58 ± 0.52 a	11.92 ± 1.01 ab	8.58 ± 0.34 b
Plant coverage	%	2661	< 0.0001 ***	84.08 ± 0.55 c	95.25 ± 0.95 a	91.83 ± 1.37 ab	52.58 ± 3.94 d	0 ± 0 e
**Pest control**								
Dominant aphid abundance	# of individuals	17.12	< 0.0001 ***	485.81 ± 34.84 ab	449.93 ± 127.98 b	315.05 ± 62.79 b	534.34 ± 31.73 ab	687.32 ± 40.86 a
Predator abundance	# of individuals	100.3	< 0.0001 ***	4.76 ± 0.54 b	4.01 ± 0.46 b	6.36 ± 0.35 a	4.44 ± 0.28 b	2.92 ± 0.2 c
Predator:Aphid ratio	ratio	24.15	< 0.0001 ***	0.0097 ± 0.0004 b	0.0142 ± 0.0064 ab	0.0217 ± 0.0025 a	0.0084 ± 0.0009 b	0.0043 ± 0.0006 b
**Pollination**								
Pollinator abundance	# of individuals	15.54	0.0037 **	21 ± 4.06 a	17.75 ± 3.09 a	13.75 ± 4.03 ab	11 ± 4.6 ab	6.25 ± 1.11 b
Fruit set	Fruit:Flower ratio	23.99	< 0.0001 ***	0.194 ± 0.013 a	0.175 ± 0.007 ab	0.18 ± 0.019 ab	0.143 ± 0.011 bc	0.127 ± 0.012 c
**Food provisioning**								
Apple yield	kg tree-1	17.41	0.0016 **	16.94 ± 2.85 ab	23.78 ± 2.73 a	19.91 ± 3 ab	13.06 ± 2 b	11.99 ± 1.96 b
Fruit weight	g	4.679	0.3219	234.35 ± 27.84	239.03 ± 30.04	199.75 ± 6.84	216.23 ± 12.85	199.97 ± 7.99
**Carbon stock**								
Soil organic carbon content	g/kg	11.98	0.0175*	2.99 ± 1.94 a	2.69 ± 1.66 ab	2.48 ± 1.03 ab	2.57 ± 2.71 ab	1.6 ± 0.39 b
Understory carbon content	g/m2	268.1	< 0.0001 ***	4.94 ± 0.24 a	3.33 ± 0.2 c	4.86 ± 0.58 a	1.84 ± 0.16 b	0 ± 0 d
**Nutrient cycling**								
**Enzymatic activity**								

β-1, 4-glucosidase	g/kg	30.46	< 0.0001 ***	50.45 ± 5.57 a	44.87 ± 1.38 ab	40.77 ± 1.78 b	43.52 ± 1.03 ab	33.88 ± 1.15 c
Alkaline phosphatase	g/m2	10.02	0.0400 *	186.96 ± 22.87 ab	214.78 ± 9.91 a	181.96 ± 42.9 ab	211.22 ± 12.5 ab	136.34 ± 8.86 b
Urease	g/kg	23.73	< 0.0001 ***	43.17 ± 3.37 bc	56.45 ± 1.08 a	47.06 ± 1.34 b	48.44 ± 1.44 b	41.73 ± 0.91 c
Peroxidase	g/m2	71	< 0.0001 ***	1.6 ± 0.11 ab	1.43 ± 0.17 b	1.88 ± 0.09 a	1.43 ± 0.08 b	1.28 ± 0.05 b
**Soil nutrient availability**								
Alkali-hydrolyzable nitrogen	mg/kg	18.79	< 0.0001 ***	75.56 ± 10.41 a	77.5 ± 3.44 a	73.19 ± 8.4 ab	48.06 ± 14.54 b	47.66 ± 4.52 b
Available phosphorus	mg/kg	20.49	< 0.0001 ***	57.78 ± 1.6 a	50.85 ± 4.17 ab	56.88 ± 1.52 a	53.84 ± 2.62 ab	45.4 ± 1.95 b
Available potassium	mg/kg	17.2	0.0018 **	83.83 ± 4.48 ab	87.38 ± 1.4 a	86.66 ± 1.15 a	87.44 ± 1.9 a	77.18 ± 1.55 b
**Leaf nutrient status**								
Leaf N content	mg/g	8.995	0.0612	28.628 ± 0.056	29.165 ± 0.005	29.045 ± 0.317	28.358 ± 0.52	28.33 ± 0.295
Leaf P content	mg/g	7.034	0.1341	3.753 ± 0.247	4.285 ± 0.329	3.625 ± 0.053	3.688 ± 0.259	3.54 ± 0.298
Leaf K content	mg/g	15.07	0.0046 **	10.763 ± 0.454 a	9.735 ± 0.404 ab	10.745 ± 0.271 a	9.845 ± 0.548 ab	9.278 ± 0.48 b

Table S3. Generalized linear mixed model (GLMM) results for individual ecosystem service (ES) indicators in each intercropped flowering plant treatment in 2021. Data are the mean ± standard error. Different lowercase letters denote significant differences ($p < 0.05$) among treatments based on Tukey's pairwise comparisons.

**ES indicator**	**Unit**	**χ^2**	**GLMM p-value**	***Brassica napus***	***Vicia villosa***	***Cnidium monnieri***	***Schizonepeta tenuifolia***	**Control**
**Habitat provisioning**								
Arthropod abundance	# of individuals	81.422	< 0.0001 ***	73.67 ± 4.91 b	62.33 ± 4.99 bc	91.67 ± 8.87 a	54.25 ± 4.85 cd	43.75 ± 4.54 d
Arthropod taxon richness	no. of taxa per plot	132.87	< 0.0001 ***	20.67 ± 0.76 b	18.67 ± 0.47 c	22.92 ± 0.82 a	18.25 ± 0.34 cd	15.5 ± 0.29 e
Plant coverage	%	7074.3	< 0.0001 ***	88.21 ± 1.45 b	92.67 ± 0.28 a	95.78 ± 0.32 a	83.91 ± 2.31 c	0 d
**Pest control**								
Dominant aphid abundance	# of individuals	43.761	< 0.0001 ***	155.43 ± 29.07 ab	131.22 ± 23.09 b	42.47 ± 15.77 c	177.65 ± 20.49 ab	238.81 ± 14.12 a
Predator abundance	# of individuals	10.197	0.0372 *	6.69 ± 0.8 ab	7.09 ± 1.71 ab	8.53 ± 0.87 a	6.41 ± 0.95 ab	2.92 ± 0.33 b
Predator:Aphid ratio	ratio	59.085	< 0.0001 ***	0.051 ± 0.015 b	0.054 ± 0.01 b	0.274 ± 0.068 a	0.039 ± 0.009 b	0.012 ± 0.001 b
**Pollination**								
Pollinator abundance	# of individuals	16.642	0.0023**	18.5 ± 6.22 a	8.75 ± 1.38 ab	13 ± 2.97 ab	7 ± 1.58 b	5.25 ± 1.89 b
Fruit set	Fruit: Flower ratio	15.21	0.0043 **	0.194 ± 0.013 a	0.185 ± 0.009 a	0.188 ± 0.034 a	0.146 ± 0.031 ab	0.111 ± 0.007 b
**Food provisioning**								
Apple yield	kg $tree^{-1}$	20.616	0.0003 ***	19.56±3.23 abc	25.03±1.69 a	23.17±2.56 ab	16.44±1.3 bc	13.99±2.36 c
Fruit weight	g	1.951	0.7448	263.25 ± 15.11	267.65 ± 4.05	263.5 ± 1.98	259.88 ± 5.82	255.54 ± 5.98
**Carbon stock**								
Soil organic carbon content	g/kg	27.698	< 0.0001 ***	15.51 ± 1.92 a	17.99 ± 2.34 a	14.73 ± 2.37 a	14.63 ± 2.23 a	8.03 ± 0.39 b
Understory carbon content	g/m^2	219.64	< 0.0001 ***	2.44 ± 0.37 b	1.6 ± 0.09 c	3.52 ± 0.28 a	0.8 ± 0.07 d	0 ± 0 e
**Nutrient cycling**								
**Enzymatic activity**								
β-1, 4-glucosidase	g/kg	71.95	< 0.0001 ***	60.22 ± 2.03 a	46.66 ± 1.44 b	46.48 ± 3.69 b	45.93 ± 2.63 b	36.36 ± 0.26 c
Alkaline phosphatase	g/m^2	131.32	< 0.0001 ***	161.48 ± 7.41 ab	179.76 ± 0.95 a	122.82 ± 7.55 c	140.31 ± 9.3 bc	93.38 ± 8.61 d

Urease	g/kg	10.252	0.0364 *	45.21 ± 3.95 a	41.57 ± 3.44 ab	42.75 ± 6.45 ab	40.82 ± 2.4 ab	31.2 ± 3.29 b
Peroxidase	g/m^2	41.878	< 0.0001 ***	1.23 ± 0.04 a	1.28 ± 0.04 a	1.09 ± 0.09 ab	0.94 ± 0.03 b	0.91 ± 0.07 b
**Soil nutrient availability**								
Alkali-hydrolyzable nitrogen	mg/kg	11.423	0.0222 *	98 ± 17.21 ab	112.88 ± 10.04 a	91.88 ± 8.85 ab	80.5 ± 8.69 ab	67.38 ± 12.81 b
Soil extractable P content	mg/kg	13.72	0.0082**	83.76 ± 8.91 a	79.84 ± 4.68 ab	82.24 ± 4.33 a	72.97 ± 4.13 ab	62.08 ± 4.1 b
Soil extractable K content	mg/kg	4.4741	0.3456	93.3 ± 2.6	92.23 ± 2.96	89.82 ± 2.95	95.97 ± 1.93	91.84 ± 4.21
**Leaf nutrient status**								
Leaf N content	mg/g	8.5874	0.0723	28.6 ± 0.92	29.55 ± 1.81	26.43 ± 0.62	28.15 ± 0.73	26.73 ± 0.52
Leaf P content	mg/g	2.5777	0.6308	2.83 ± 0.33	2.53 ± 0.28	2.48 ± 0.27	2.43 ± 0.06	2.45 ± 0.1
Leaf K content	mg/g	10.925	0.0274 *	8.43 ± 1.23 ab	9.28 ± 1.2 ab	9.78 ± 0.8 a	7.29 ± 0.49 ab	6.8 ± 0.29 b

Table S4. Results of statistical analysis of six ecosystem service composite indicators by each treatment in 2020. Table includes the mean and standard error (SE) per treatment, P value and F value. Different letters denote significant differences (p < 0.05) between treatments based on Tukey pairwise comparisons.

ES Composite Index	F-value	DF	P-value	*Brassica napus*	*Vicia villosa*	*Cnidium monnieri*	*Schizonepeta tenuifolia*	Control
Pest Control	6.276	4	0.00357 **	0.46 ± 0.01bc	0.52 ± 0.18 ab	0.8 ± 0.07 a	0.4 ± 0.04 bc	0.18 ± 0.04 c
Food Provisioning	3.407	4	0.0359 *	0.43 ± 0.06 ab	0.63 ± 0.13 a	0.44 ± 0.08 ab	0.29 ± 0.07 b	0.23 ± 0.07 b
Pollination	4.561	4	0.0131 *	0.75 ± 0.10 a	0.61 ± 0.08 ab	0.54 ± 0.15 ab	0.35 ± 0.11 bc	0.19 ± 0.04 c
Carbon Stock	6.139	4	0.00393 **	0.78 ± 0.10 a	0.62 ± 0.10 a	0.64 ± 0.03 a	0.52 ± 0.16 a	0.16 ± 0.02 b
Habitat Provisioning	26.7	4	< 0.001 ***	0.73 ± 0.05 b	0.69 ± 0.08 b	0.94 ± 0.02 a	0.58 ± 0.06 b	0.19 ± 0.03 c
Nutrient Cycling	4.044	4	0.0202 *	0.66 ± 0.17 a	0.70 ± 0.03 a	0.54 ± 0.03 a	0.55 ± 0.08 a	0.24 ± 0.05 b

Table S5. Results of statistical analysis of six ecosystem service composite indicators by each treatment in 2021. Table includes the mean and standard error (SE) per treatment, P value and F value. Different letters denote significant differences (p < 0.05) between treatments based on Tukey pairwise comparisons.

ES Composite Index	F-value	DF	P-value	*Brassica napus*	*Vicia villosa*	*Cnidium monnieri*	*Schizonepeta tenuifolia*	Control
Pest Control	18.92	4	< 0.001 ***	0.38 ± 0.06 b	0.43 ± 0.04 b	0.83 ± 0.09 a	0.33 ± 0.05 bc	0.15 ± 0.02 c
Food Provisioning	3.21	4	0.0432 *	0.6 ± 0.2 abc	0.85 ± 0.08 a	0.75 ± 0.11 ab	0.45 ± 0.08 bc	0.32 ± 0.1 c
Pollination	3.576	4	0.0307 *	0.61 ± 0.15 a	0.39 ± 0.02 abc	0.48 ± 0.08 ab	0.29 ± 0.07 bc	0.21 ± 0.04 c
Carbon Stock	7.757	4	0.00135 **	0.66 ± 0.09 a	0.56 ± 0.07 a	0.88 ± 0.07 a	0.48 ± 0.1 a	0.16 ± 0.12 b
Habitat Provisioning	42.78	4	< 0.001 ***	0.69 ± 0.06 c	0.56 ± 0.03 c	0.88 ± 0.05 a	0.48 ± 0.02 c	0.16 ± 0.03 d
Nutrient Cycling	19.96	4	< 0.001 ***	0.74 ± 0.1 a	0.66 ± 0.08 b	0.44 ± 0.03 b	0.45 ± 0.03 b	0.19 ± 0.04 c

Table S6. Taxonomic units of arthropods recorded in 2020 after taxonomic cleaning, with identification levels used for arthropod taxon-richness calculations. This table provides the taxonomic units used for arthropod taxon-richness calculations. Treatment-level quantitative data for arthropod abundance and arthropod taxon richness are presented in Tables A.2 and A.3. Because not all specimens could be reliably identified to species level, richness was reported as taxon richness rather than strict species richness.

**No.**	**Taxon used in analysis**	**Scientific name / taxonomic unit**	**Order / family**	**Identification level**
1	Black-banded syrphid fly	*Episyrphus balteatus*	Diptera, Syrphidae	Species
2	Asian ladybeetle	*Harmonia axyridis*	Coleoptera, Coccinellidae	Species
3	Tortoise-patterned ladybeetle	*Propylea japonica*	Coleoptera, Coccinellidae	Species
4	Variegated ladybeetle	*Hippodamia variegata*	Coleoptera, Coccinellidae	Species
5	Green lacewing	*Chrysopidae sp.*	Neuroptera, Chrysopidae	Family / morphotaxon
6	Three-spotted crab spider	*Ebrechtella tricuspidata*	Araneae, Thomisidae	Species
7	Aphidophagous mirid bug	*Deraeocoris sp.*	Hemiptera, Miridae	Genus / morphotaxon
8	Large grey syrphid fly	*Eupeodes corollae*	Diptera, Syrphidae	Species
9	Unidentified syrphid fly	*Syrphidae sp.*	Diptera, Syrphidae	Family / morphotaxon
10	White-margined leafhopper	*Cicadellidae morphospecies 1*	Hemiptera, Cicadellidae	Family / morphospecies
11	Grey leafhopper	*Cicadellidae morphospecies 2*	Hemiptera, Cicadellidae	Family / morphospecies
12	Green leafhopper	*Cicadellidae morphospecies 3*	Hemiptera, Cicadellidae	Family / morphospecies
13	Unidentified muscid fly	*Muscidae sp.*	Diptera, Muscidae	Family / morphotaxon
14	Unidentified mirid bug 1	*Miridae morphospecies 1*	Hemiptera, Miridae	Family / morphospecies
15	Diamondback moth	*Plutella xylostella*	Lepidoptera, Plutellidae	Species
16	Red-legged patterned fly	*Diptera morphospecies 1*	Diptera	Order / morphospecies

17	Willow leaf-miner fly	*Agromyzidae sp.*	Diptera, Agromyzidae	Family / morphotaxon
18	Tumbling flower beetle	*Mordellidae sp.*	Coleoptera, Mordellidae	Family / morphotaxon
19	Unidentified lepidopteran	*Lepidoptera morphospecies 1*	Lepidoptera	Order / morphospecies
20	Honey bee	*Apidae sp.*	Hymenoptera, Apidae	Family / morphotaxon
21	Long-abdomen syrphid fly	*Syrphidae morphospecies 1*	Diptera, Syrphidae	Family / morphospecies
22	Unidentified spider	*Araneae morphospecies 1*	Araneae	Order / morphospecies
23	Striped flea beetle	*Phyllotreta striolata*	Coleoptera, Chrysomelidae	Species
24	Unidentified mirid bug 2	*Miridae morphospecies 2*	Hemiptera, Miridae	Family / morphospecies
25	Zygaenid moth	*Zygaenidae sp.*	Lepidoptera, Zygaenidae	Family / morphotaxon
26	Short-abdomen syrphid fly	*Syrphidae morphospecies 2*	Diptera, Syrphidae	Family / morphospecies
27	Big-eyed bug	*Geocoridae sp.*	Hemiptera, Geocoridae	Family / morphotaxon
28	Red-striped stink bug	*Graphosoma rubrolineatum**	Hemiptera, Pentatomidae	Species
29	Cabbage white butterfly	*Pieris rapae*	Lepidoptera, Pieridae	Species
30	Ichneumonid wasp	*Ichneumonidae sp.*	Hymenoptera, Ichneumonidae	Family / morphotaxon
31	Tortricid moth	*Tortricidae sp.*	Lepidoptera, Tortricidae	Family / morphotaxon
32	Minute pirate bug	*Orius sp.*	Hemiptera, Anthocoridae	Genus / morphotaxon
33	Blow fly	*Calliphoridae sp.*	Diptera, Calliphoridae	Family / morphotaxon

Table S7. Taxonomic units of arthropods recorded in 2021 after taxonomic cleaning, with identification levels used for arthropod taxon-richness calculations. This table provides the taxonomic units used for arthropod taxon-richness calculations. Treatment-level quantitative data for arthropod abundance and arthropod taxon richness are presented in Tables A.2 and A.3. Because not all specimens could be reliably identified to species level, richness was reported as taxon richness rather than strict species richness.

No.	Taxon used in analysis	Scientific name / taxonomic unit	Order / family	Identification level
1	Black-banded syrphid fly	*Episyrphus balteatus*	Diptera, Syrphidae	Species
2	Red-legged patterned fly	Diptera morphotaxon 1	Diptera	Order / morphotaxon
3	Tortoise-patterned ladybeetle	*Propylea japonica*	Coleoptera, Coccinellidae	Species
4	Green mirid bug	*Apolygus lucorum*	Hemiptera, Miridae	Species
5	Asian ladybeetle	*Harmonia axyridis*	Coleoptera, Coccinellidae	Species
6	Brassica stink bug	Eurydema sp.	Hemiptera, Pentatomidae	Genus / morphotaxon
7	Short-horned mirid bug	*Polymerus brevicornis*	Hemiptera, Miridae	Species
8	Green lacewing	*Chrysopa pallens*	Neuroptera, Chrysopidae	Species
9	Italian honey bee	*Apis mellifera ligustica*	Hymenoptera, Apidae	Subspecies
10	Holarctic brown lacewing	*Hemerobius humulinus*	Neuroptera, Hemerobiidae	Species
11	Chinese leaf beetle	*Chrysochus chinensis*	Coleoptera, Chrysomelidae	Species
12	Red-bellied hymenopteran	Hymenoptera morphotaxon 1	Hymenoptera	Order / morphotaxon
13	Striped flea beetle	*Phyllotreta striolata*	Coleoptera, Chrysomelidae	Species
14	Short-tubed syrphid fly	Syrphidae morphotaxon 1	Diptera, Syrphidae	Family / morphotaxon
15	Large grey syrphid fly	*Eupeodes corollae*	Diptera, Syrphidae	Species
16	Potato ladybird beetle	*Henosepilachna vigintioctomaculata*	Coleoptera, Coccinellidae	Species
17	Asparagus carpenter moth	Cossidae morphotaxon 1	Lepidoptera, Cossidae	Family / morphotaxon
18	Dance fly	Empididae sp.	Diptera, Empididae	Family / morphotaxon
19	Small spotted-wing fly morphotaxon	Diptera morphotaxon 2	Diptera	Order / morphotaxon

20	Green bottle fly	*Lucilia sericata*	Diptera, Calliphoridae	Species
21	Striped red-whiskered mirid bug	*Trigonotylus caelestialium*	Hemiptera, Miridae	Species
22	Long-tubed syrphid fly	Syrphidae morphotaxon 2	Diptera, Syrphidae	Family / morphotaxon
23	Pasture mirid bug	*Lygus pratensis*	Hemiptera, Miridae	Species
24	Red-striped stink bug	*Graphosoma rubrolineatum*	Hemiptera, Pentatomidae	Species
25	Three-spotted crab spider	*Ebrechtella tricuspidata*	Araneae, Thomisidae	Species
26	American serpentine leaf miner	*Liriomyza sativae*	Diptera, Agromyzidae	Species
27	Jewel beetle morphotaxon	Buprestidae morphotaxon 1	Coleoptera, Buprestidae	Family / morphotaxon
28	Chalcidoid parasitoid of bark beetles	Chalcidoidea morphotaxon 1	Hymenoptera, Chalcidoidea	Superfamily / morphotaxon
29	Dark-spotted syrphid fly	Syrphidae morphotaxon 3	Diptera, Syrphidae	Family / morphotaxon
30	Metallic green chalcidoid wasp	Chalcidoidea morphotaxon 2	Hymenoptera, Chalcidoidea	Superfamily / morphotaxon
31	Varied carpet beetle	Dermestidae morphotaxon 1	Coleoptera, Dermestidae	Family / morphotaxon
32	Hornet / paper wasp morphotaxon	Vespidae morphotaxon 1	Hymenoptera, Vespidae	Family / morphotaxon
33	Chinese sawfly	Symphyta morphotaxon 1	Hymenoptera, Symphyta	Suborder / morphotaxon
34	Asian corn borer	*Ostrinia furnacalis*	Lepidoptera, Crambidae	Species
35	Three-spotted alfalfa mirid bug	*Adelphocoris fasciaticollis*	Hemiptera, Miridae	Species
36	Japanese horned powder-post beetle	*Sinoxylon japonicum*	Coleoptera, Bostrichidae	Species
37	Brown marmorated stink bug	*Halyomorpha halys*	Hemiptera, Pentatomidae	Species
38	Chestnut longhorn beetle	Cerambycidae morphotaxon 1	Coleoptera, Cerambycidae	Family / morphotaxon
39	Millet alydid / coreid bug	Alydidae/Coreidae morphotaxon 1	Hemiptera, Alydidae/Coreidae	Family / morphotaxon
40	Bean bug	*Riptortus pedestris*	Hemiptera, Alydidae	Species
41	Ziziphus leaf beetle	Chrysomelidae morphotaxon 1	Coleoptera, Chrysomelidae	Family / morphotaxon
42	Chinese horse fly	Tabanidae morphotaxon 1	Diptera, Tabanidae	Family / morphotaxon
43	Japanese paper wasp	*Polistes japonicus*	Hymenoptera, Vespidae	Species
44	Ichneumonid parasitoid of gypsy moth	*Pimpla disparis*	Hymenoptera, Ichneumonidae	Species
45	Lepidopteran morphotaxon	Lepidoptera morphotaxon 1	Lepidoptera	Order / morphotaxon

46	Zygaenid moth	Zygaenidae sp.	Lepidoptera, Zygaenidae	Family / morphotaxon
47	Ichneumonid parasitoid of syrphid flies	Ichneumonidae morphotaxon 1	Hymenoptera, Ichneumonidae	Family / morphotaxon

Table S8. Mean abundance (± SE) of the most abundant insect taxa under different flowering-intercrop treatments in 2020 and 2021. Values are presented as means ± SE based on four replicate plots. Pest and natural-enemy taxa were identified to species level, whereas bees were identified and quantified collectively at the superfamily level as Apoidea. The taxa presented in this table were the most abundant taxa within their respective functional groups across the two study years.

**Functional group**	**Taxon**	**Taxonomic level**	**Year**	***Brassica napus***	***Vicia villosa***	***Cnidium monnieri***	***Schizonepeta tenuifolia***	**Control**
Pest	*Aphis spiraecola*	Species	2020	485.81 ± 34.84	449.93 ± 127.98	315.05 ± 62.79	534.34 ± 31.73	687.32 ± 40.86
			2021	155.43 ± 29.07	131.22 ± 23.09	42.47 ± 15.77	177.65 ± 20.49	238.81 ± 14.12
Natural enemy	*Harmonia axyridis*	Species	2020	3.42 ± 0.24	2.69 ± 0.3	4.19 ± 0.26	3.09 ± 0.2	2.24 ± 0.09
			2021	3.6 ± 0.2	3.55 ± 0.81	4.25 ± 0.28	3.48 ± 0.52	2.34 ± 0.17
Natural enemy	*Chrysoperla sinica*	Species	2020	0.24 ± 0.25	0.3 ± 0.23	0.26 ± 0.16	0.2 ± 0.13	0.09 ± 0.06
			2021	2.82 ± 0.73	2.94 ± 0.76	3.39 ± 0.8	2.77 ± 0.53	1.39 ± 0.17
Natural enemy	*Episyrphus balteatus*	Species	2020	0.25 ± 0.04	0.23 ± 0.04	0.16 ± 0.1	0.13 ± 0.05	0.06 ± 0.05
			2021	0.09 ± 0.04	0.42 ± 0.26	0.26 ± 0.21	0.06 ± 0.04	0.08 ± 0.02
Natural enemy	*Ebrechtella tricuspidata*	Species	2020	0.04 ± 0.02	0.04 ± 0.02	0.1 ± 0.02	0.05 ± 0.03	0.05 ± 0.01
			2021	0.18 ± 0.05	0.19 ± 0.05	0.13 ± 0.07	0.09 ± 0.02	0.11 ± 0.012
Pollinator	Apoidea	Superfamily	2020	8.75 ± 2.32	7 ± 1.22	5 ± 1.08	1 ± 0.41	3.5 ± 1.55
			2021	15.75 ± 6.14	33.5 ± 5.78	7 ± 1.87	5.75 ± 1.93	5.25 ± 1.89
